# Endothelial Activation and Permeability in Patients on VV-ECMO Support: An Exploratory Study

**DOI:** 10.3390/jcm14144866

**Published:** 2025-07-09

**Authors:** Carolien Volleman, Yakun Li, Anita M. Tuip-de Boer, Chantal A. Polet, Roselique Ibelings, Marleen A. Slim, Henrike M. Hamer, Alexander P. J. Vlaar, Charissa E. van den Brom

**Affiliations:** 1 Department of Intensive Care Medicine, Amsterdam UMC, University of Amsterdam, 1105 AZ Amsterdam, The Netherlands; c.volleman@amsterdamumc.nl (C.V.);; 2 Laboratory for Experimental Intensive Care and Anesthesiology (LEICA), Amsterdam UMC, University of Amsterdam, 1105 AZ Amsterdam, The Netherlands; 3 Department of Anesthesiology, Amsterdam UMC, VU University, 1081 HV Amsterdam, The Netherlands; 4 Center for Experimental and Molecular Medicine, Amsterdam UMC, Amsterdam Institute for Infection and Immunity, University of Amsterdam, 1105 AZ Amsterdam, The Netherlands; 5 Laboratory Specialized Diagnostics & Research, Department of Laboratory Medicine, Amsterdam UMC, University of Amsterdam, 1105 AZ Amsterdam, The Netherlands

**Keywords:** extracorporeal membrane oxygenation, ECMO, ARDS, endothelium, endothelial activation, inflammation, hemolysis

## Abstract

**Background** Veno-venous extracorporeal membrane oxygenation (VV-ECMO) supports critically ill patients with respiratory failure. However, ECMO may induce systemic inflammation, hemolysis, and hemodilution, potentially resulting in endothelial activation and damage. Therefore, this study explored the longitudinal changes in circulating markers of inflammation, hemolysis, and endothelial activation and damage in patients with COVID-19 on VV-ECMO. **Methods** Plasma was obtained before, within 48 h as well as on day 4, week 1, and week 2 of ECMO support and after decannulation. Circulating markers were measured using Luminex, ELISA, and spectrophotometry. Human pulmonary endothelial cells were exposed to patient plasma, and in vitro endothelial permeability was assessed using electric cell-substrate impedance sensing. **Results** From April 2020 to January 2022, plasma was collected from 14 patients (71.4% male; age 54 (45–61) years). IL-6 levels decreased (1.238 vs. 0.614 ng/mL, *p* = 0.039) while ICAM-1 increased (667 vs. 884 ng/mL, *p* = 0.003) over time when compared to pre-ECMO. Angiopoietin-1 decreased after ECMO initiation (7.57 vs. 3.58 ng/mL, *p* = 0.030), whereas angiopoietin-2 increased (5.20 vs. 10.19 ng/mL, *p* = 0.017), particularly in non-survivors of ECMO. Cell-free hemoglobin decreased directly after VV-ECMO initiation but remained stable thereafter (55.29 vs. 9.19 mg/dL, *p* = 0.017). Moreover, the plasma obtained at several time points during the ECMO run induced in vitro pulmonary endothelial hyperpermeability. **Conclusions** This exploratory study shows that patients on VV-ECMO support due to COVID-ARDS exhibit progressive endothelial activation and damage but not inflammation and hemolysis. Larger prospective studies are necessary to elucidate pathophysiological pathways leading to endothelial activation and damage, thereby reducing organ failure in these critically ill patients.

## 1. Introduction

Extracorporeal membrane oxygenation (ECMO) supports critically ill patients with respiratory failure. Veno-venous ECMO (VV-ECMO) aims to ensure adequate oxygen delivery and carbon dioxide removal while the lungs are given time to recover. Despite advancements in the technique, ECMO remains associated with complications, such as hemorrhage and thromboembolic events as well as organ failure [1]. The underlying mechanisms driving these complications, including organ edema, are not fully understood, and effective prevention strategies therefore remain elusive.

ECMO induces a systemic inflammatory response due to blood contact with foreign surfaces of the circuit. This pro-inflammatory state results in complex interactions with the endothelium [2]. The generation of inflammatory mediators can activate endothelial cells and may eventually increase endothelial permeability, with leakage of fluid to the interstitium and ensuing tissue edema [2,3]. Previously, we demonstrated that extracorporeal circulation induced fluid leakage and organ edema in healthy rats [4]. Moreover, the plasma from patients undergoing cardiac surgery on cardiopulmonary bypass induced in vitro endothelial hyperpermeability [5]. Nonetheless, the clinical evidence on endothelial permeability in patients supported by ECMO is limited [6].

In addition to systemic inflammation, ECMO induces hemodilution and hemolysis, both contributing to endothelial damage [7,8]. Priming of the ECMO circuit is required leading to the hemodilution in the patient. Moreover, patients on ECMO support can be vasoplegic, due to the underlying condition as well as to the systemic inflammatory response to the ECMO circuit. To enable sufficient blood flow for the system to run, fluid resuscitation is often necessary. This fluid resuscitation can aggravate organ injury, presumably by increasing fluid leakage and tissue edema [9]. Furthermore, hemolysis occurs in 15–21% of patients supported by ECMO, primarily due to the mechanical trauma inflicted on the red blood cells in the circuit [10,11]. Intravascular hemolysis is characterized by an increase in cell-free hemoglobin (CFHb) due to the rupture of the erythrocyte membranes. Under physiological conditions, CFHb is scavenged by haptoglobin, effectively mitigating its toxic effects. However, haptoglobin levels become depleted, permitting CFHb to circulate freely and react with endothelium-derived nitric oxide, causing vasoconstriction and the formation of reactive oxygen species [12]. Recently, it has been suggested that CFHb can also activate the endothelium and increase endothelial permeability directly [8,13,14].

Endothelial damage is a less-recognized complication of ECMO. Reviews by our group have highlighted the lack of knowledge concerning the role of endothelial activation and damage patients on ECMO [6,15]. Therefore, the aim of this hypothesis-generating study was to explore the alterations in the circulating markers of systemic inflammation, hemolysis, and endothelial activation and damage in patients on VV-ECMO, and their potential impact on in vitro endothelial permeability. By assessing the changes in the circulating markers of endothelial activation and hemolysis during ECMO, this exploratory study sought to enhance the understanding of these processes and their implications for patient outcomes.

## 2. Materials and Methods

### 2.1. Patient Samples

Patient plasma samples were obtained from the Amsterdam UMC COVID Biobank. The Amsterdam UMC COVID Biobank contains plasma samples from a large prospective cohort of all confirmed patients with COVID-19 admitted to the Amsterdam UMC. The institutional review board and the biobank ethics committee of the Amsterdam UMC approved the biobank study protocol (protocol number 2021_0204). This study was conducted in accordance with the ethical standards of the ethical review board of the Amsterdam UMC and the Declaration of Helsinki of 1975. The present study cohort included 14 patients receiving VV-ECMO and 28 patients on mechanical ventilation (MV) who were admitted to intensive care between March 2020 and October 2021. The MV group comprised patients with COVID-ARDS who were mechanically ventilated and admitted to the ICU at the time of hospital admission. Plasma from 5 healthy volunteers was included as control. Plasma was obtained before initiation of ECMO, during ECMO on day 2, day 4, week 1, and week 2, as well as after decannulation. Blood was collected in heparin tubes and centrifuged at 4000× *g* for 10 min at 4 °C to obtain platelet-free plasma. Plasma supernatant was centrifuged for another 5 min at 12,000× *g* at 4 °C and stored at −80 °C.

### 2.2. Plasma Analyses

Plasma was analyzed for markers of inflammation, glycocalyx damage, endothelial injury, and endothelial damage using Luminex or enzyme-linked immunosorbent assay (ELISA). Measurements were performed according to the manufacturer. Spectrophotometry was used to assess parameters of hemolysis. An extended description of the analyses can be found in the supplementary methods (Supplementary File S1).

### 2.3. In Vitro Endothelial Permeability

Human pulmonary microvascular endothelial cells (HMVEC-L, CC-2527, Lonza Group, Basel, Switzerland) were cultured on gelatin-coated flasks in complete medium. Passage six cells were seeded on electric cell-substrate impedance sensing (ECIS; Applied BioPhysics, Troy, NY, USA) plates and exposed to 10% platelet-free patient plasma. Endothelial resistance was measured at 4000 Hz as previously described [16,17,18]. An extended description of the measurement can be found in the supplementary methods (Supplementary File S1).

### 2.4. Statistical Analysis

A formal sample size calculation was not performed due to the exploratory study design. Instead, we aimed to include all patients on VV-ECMO with available plasma samples from our Amsterdam UMC COVID-19 Biobank, ensuring the maximum use of data.

Propensity score matching was performed to align the MV group with our cohort of patients on ECMO, based on gender and age (1:2 ratio, caliper 0.2 standard deviations). To assess the covariate balance in a sample-size-independent manner, we evaluated the absolute value of the standardized mean difference (SMD) in the unmatched and the propensity-score-matched samples using the R cobalt package (version 4.3.2, Supplementary File S2). An SMD within the range of 0 to 0.2 was considered in balance.

Continuous data are presented as mean ± standard deviation for normally distributed data or median with interquartile range for non-parametric data. The normality of the data was checked via Q-Q plots and Shapiro–Wilk tests. Changes in circulating markers and endothelial resistance over time in patients on ECMO were analyzed using linear mixed-effects (LME) models with Tukey post hoc analyses. Furthermore, LME analyses were performed to assess differences over time between survivors and non-survivors of ECMO. Differences between patients on ECMO and MV were analyzed using Chi-square tests or Mann–Whitney U tests. *p* values < 0.05 were considered as statistically significant. Data were analyzed using R-Studio (version 4.2.3; Boston, MA, USA), and graphs were created using GraphPad Prism 10 (GraphPad Software, San Diego, CA, USA).

## 3. Results

### 3.1. Patient Characteristics

Patient demographics and clinical parameters are shown in Table 1. Between March 2020 and October 2021, a total of 14 patients on VV-ECMO were included in the Amsterdam UMC COVID Biobank. Additionally, 28 patients with COVID-ARDS who were mechanically ventilated were selected after matching. The majority of patients were male (ECMO 71%, MV 79%), and the median age was 54 (45–61) years in the ECMO group and 54 (50–62) years in the MV group. Furthermore, hypertension and diabetes mellitus type 2 were equally present in both groups. SOFA scores were 8 (5–9) in the ECMO group and 5 (4–8) in the patients in the MV group. The overall in-hospital mortality in the entire cohort was 38%.

### 3.2. Circulating Markers in Patients on VV-ECMO over Time

**Figure 1:**
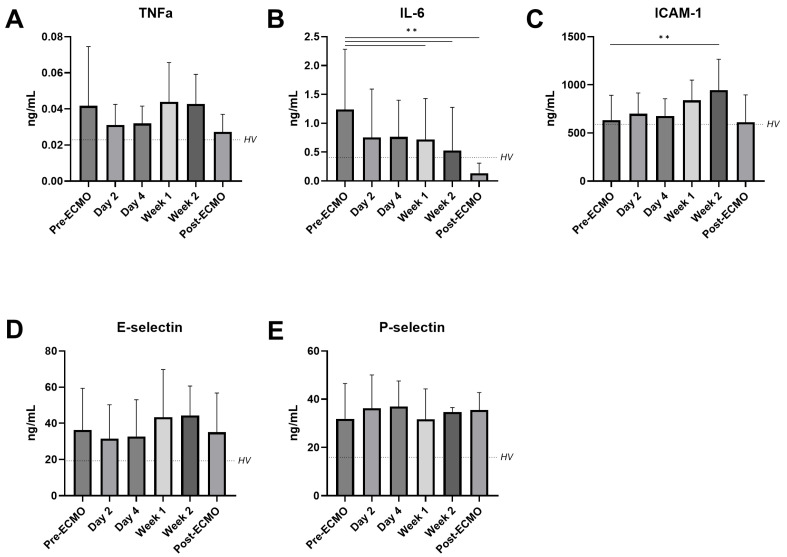
Circulating markers of inflammation and endothelial dysfunction in patients receiving VV-ECMO. Circulating tumor necrosis factor α (TNFα (**A**)), interleukin 6 (IL-6 (**B**)), intercellular adhesion molecule 1 (ICAM-1 (**C**)), E-selectin (**D**), and P-selectin (**E**) in plasma from patients on VV-ECMO obtained before initiation of ECMO (pre-ECMO); on day 2, day 4, week 1, and week 2 of ECMO support; and after decannulation (post-ECMO). The dotted line represents the mean values in healthy volunteers (HV). Data are presented as mean with standard deviation and were tested using mixed-effects models. ** *p* ≤ 0.01.

Firstly, the circulating markers of inflammation and endothelial activation were measured. Patients supported by ECMO showed decreasing levels of IL-6 when compared to pre-ECMO (1.238 vs. 0.614 ng/mL (week 2), *p* = 0.039; Figure 1B), and even a further decline after decannulation (1.238 vs. 0.460 ng/mL, *p* = 0.026). The circulating levels of TNF-α remained stable while on ECMO. In contrast, the levels of ICAM-1 increased during their ECMO run (pre-ECMO 667 ng/mL vs. week 1 884 ng/mL, *p* = 0.003; Figure 1A). The plasma levels of E-selectin and P-selectin did not change over time in patients supported by ECMO (Figure 1D,E).

**Figure 2:**
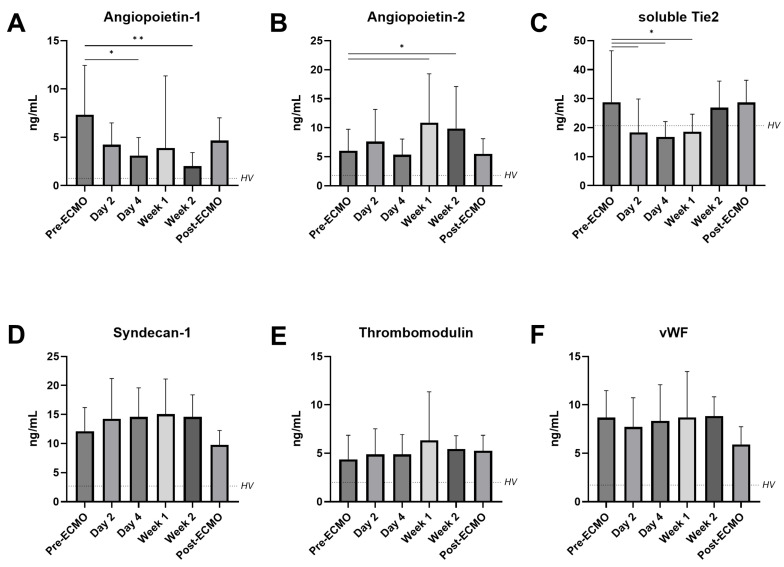
**Circulating markers of endothelial damage and glycocalyx degradation in patients on VV-ECMO.** Circulating angiopoietin-1 (**A**), angiopoietin-2 (**B**), soluble Tie2 (**C**), syndecan-1 (**D**), thrombomodulin (**E**), and von Willebrand Factor (vWF (**F**)) in plasma from patients on VV-ECMO obtained before initiation of ECMO (pre-ECMO); on day 2, day 4, week 1, and week 2 of ECMO support; and after decannulation (post-ECMO). The dotted line represents the mean values in healthy volunteers (HV). Data are presented as mean with standard deviation and were tested using mixed-effects models. * *p* ≤ 0.05, ** *p* ≤ 0.01.

Secondly, the markers of glycocalyx degradation and endothelial damage were assessed. The angiopoietin-1 concentration decreased after four days of ECMO and continued to decrease until week 2 when compared to pre-ECMO (respectively 7.57 vs. 3.58 ng/mL, *p* = 0.030; 7.57 vs. 1.93 ng/mL, *p* = 0.0074; Figure 2A). On the contrary, the circulating levels of angiopoietin-2 increased after the initiation of ECMO (pre-ECMO 5.20 vs. week 1 10.19 ng/mL, *p* = 0.017; Figure 2B). Soluble Tie2 (sTie2) decreased during the first week of ECMO but seemed to stabilize thereafter (pre-ECMO 28.1 vs. week 1 18.5 ng/mL, *p* = 0.046; Figure 2C). The plasma levels of syndecan-1, thrombomodulin, and von Willebrand factor (vWF) did not show a significant change over time (Figure 2D–F).

**Figure 3:**
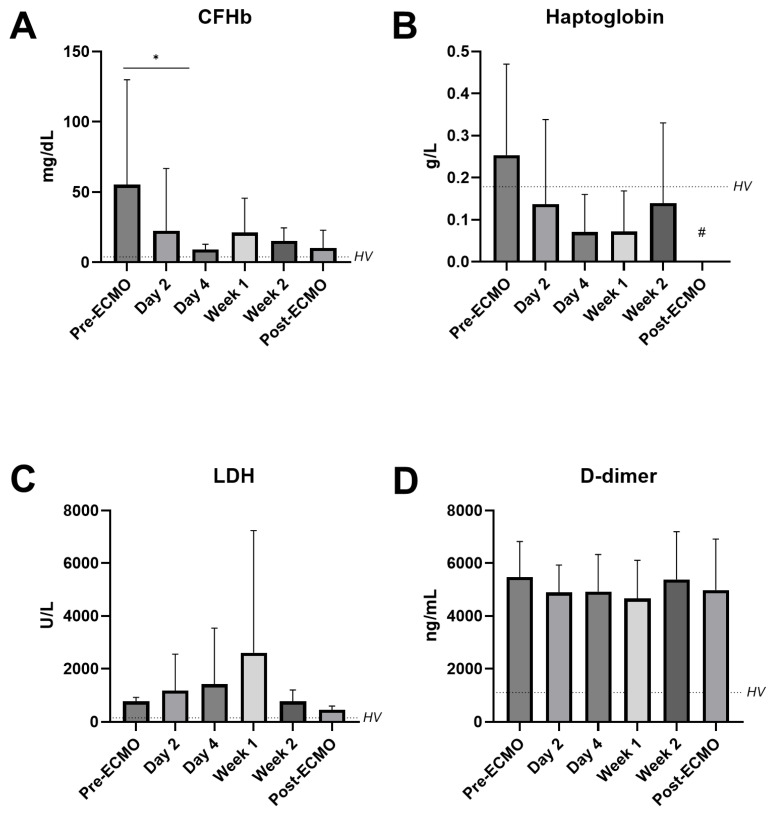
**Circulating markers of hemolysis and hemostasis in patients on VV-ECMO.** Circulating cell-free hemoglobin (CFHb (**A**)), haptoglobin (**B**), lactate dehydrogenase (LDH (**C**)), and D-dimer (**D**) in plasma from patients on VV-ECMO obtained before initiation of ECMO (pre-ECMO); on day 2, day 4, week 1, and week 2 of ECMO support; and after decannulation (post-ECMO). The dotted line represents the mean values in healthy volunteers (HVs). Data are present as mean with standard deviation and were tested using mixed-effects models. * *p* ≤ 0.05, # not enough sample available per patient.

Thirdly, the markers of hemolysis and hemostasis during VV-ECMO are presented in Figure 3. The CFHb levels in plasma were highest prior to ECMO and decreased after initiation (pre-ECMO 55.29 vs. day 4 9.19 mg/dL, *p* = 0.017; Figure 3A). Haptoglobin, lactate dehydrogenase (LDH), and D-dimer did not change over time when compared to the levels prior to ECMO initiation (Figure 3B–D).

Lastly, the levels of circulating markers prior to ECMO were compared to the levels in the MV group (Supplementary File S3). The von Willebrand factor and D-dimer levels were higher in the patients needing ECMO compared to matched patients on MV (8.79 vs. 6.94 ng/mL, *p* = 0.044; 5732 vs. 3369 ng/mL, *p* = 0.022 respectively). None of the other markers were different prior to the initiation of ECMO.

### 3.3. Circulating Markers in Survivors and Non-Survivors

**Figure 4:**
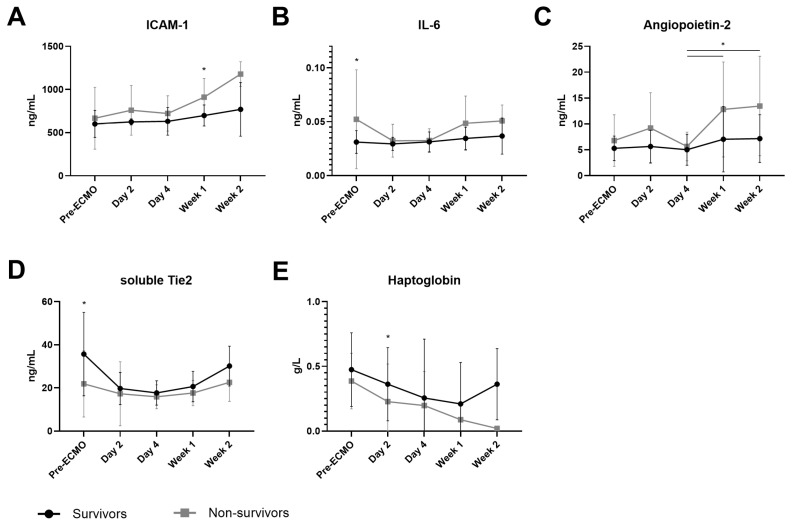
**Circulating markers in survivors and non-survivors of VV-ECMO.** Circulating intercellular adhesion molecule 1 (ICAM-1 (**A**)), interleukin 6 (IL-6 (**B**)), angiopoietin-2 (**C**), soluble Tie2 (**D**) and haptoglobin (**E**) in plasma from survivors and non-survivors of VV-ECMO obtained before initiation of ECMO (pre-ECMO), on day 2, day 4, week 1, and week 2 of ECMO support. Data represent mean with standard deviation and were tested using mixed-effects models. * *p* ≤ 0.05.

Mortality was defined as in-hospital death. Patients in our cohort who did not survive showed increasingly higher levels of ICAM-1, especially in week 1, when compared to survivors (899 vs. 578 ng/mL, *p* = 0.037; Figure 4A). Furthermore, survivors showed lower levels of IL-6 at baseline (0.711 vs. 1.713 ng/mL, *p* = 0.046; Figure 4B). The opposite was observed concerning sTie2 at baseline, which was higher in survivors compared to non-survivors (35.9 vs. 20.6 ng/L, *p* = 0.047; Figure 4D). In addition, the circulating levels of angiopoietin-1 were not different between survivors and non-survivors (Supplementary File S4). However, angiopoietin-2 increased in non-survivors after four days of ECMO (day 4 3.74 vs. week 1 11.87 ng/mL, *p* = 0.013; day 4 3.74 vs. week 2 14.27 ng/mL, *p* = 0.028; Figure 4C) but not in survivors of VV-ECMO. Syndecan-1 and thrombomodulin were not different between survivors and non-survivors (Supplementary File S4). Lastly, the change in haptoglobin was significantly different between survivors and non-survivors. Especially after two days of ECMO, survivors showed higher levels of haptoglobin compared to patients who died (0.271 vs. 0.0302 g/L, *p* = 0.044; Figure 4E). Nonetheless, the levels of CFHb and LDH were similar between the groups (Supplementary File S4).

### 3.4. Plasma from VV-ECMO Patients Induced In Vitro Endothelial Hyperpermeability

**Figure 5:**
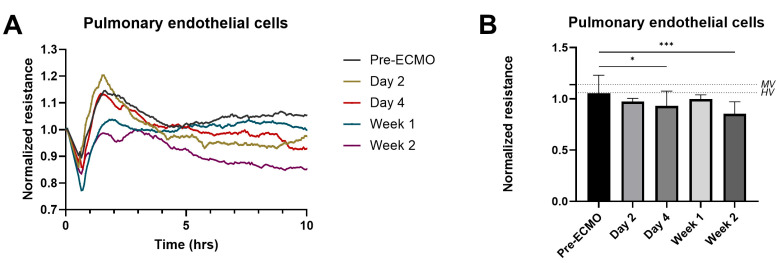
**Plasma from patients on VV-ECMO with induced in vitro pulmonary endothelial hyperpermeability.** Human pulmonary endothelial cells were exposed to plasma from critically ill patients obtained before VV-ECMO initiation (pre-ECMO), as well as after initiation of ECMO on day 2, day 4, week 1, and week 2 (**A**). Normalized resistance at 10 h of stimulation was used to assess differences between different time points (**B**). The dotted lines represent the normalized resistance using plasma from patients who were mechanically ventilated (MV) and healthy volunteers (HVs). Data are presented as mean with standard deviation and were tested using mixed-effects models. * *p* ≤ 0.05, *** *p* ≤ 0.001.

Due to systemic inflammation, patients on VV-ECMO are at increased risk of vascular leakage and edema formation. To investigate whether circulating factors in the plasma might induce endothelial hyperpermeability, we exposed pulmonary endothelial cells to patient plasma obtained at different time points during the ECMO run. We found that plasma obtained after four days of ECMO showed a lower pulmonary endothelial resistance when compared to endothelial cells exposed to plasma obtained before ECMO initiation (respectively 0.958 vs. 1.088 Ω, *p* = 0.025; Figure 5). Endothelial resistance was even lower when endothelial cells were exposed to plasma from patients after two weeks of ECMO (0.864 vs. 1.088 Ω, *p* < 0.001). In vitro pulmonary endothelial resistance did not differ between plasma from patients prior to ECMO and MV.

## 4. Discussion

This exploratory study aimed to assess changes in circulating markers of inflammation, hemolysis, endothelial activation, and endothelial damage in patients before, during, and after VV-ECMO support. In addition, we explored whether these alterations in the circulating markers from these patients affected in vitro endothelial permeability. Our data show that patients on VV-ECMO support had increased endothelial activation and damage over time but not inflammation. Surprisingly, the levels of CFHb were highest prior to ECMO initiation but remained stable thereafter. Additionally, no significant changes over time were observed concerning other markers of hemolysis. Interestingly, early differences in markers of inflammation and endothelial damage could distinguish survivors from non-survivors during VV-ECMO. In addition, we demonstrate that the plasma from patients on VV-ECMO support leads to in vitro pulmonary endothelial hyperpermeability, with this effect becoming more pronounced using plasma obtained at later time points during the ECMO run. Taken together, these results suggest that while VV-ECMO support may not drive further inflammation or hemolysis in these patients, it does appear to exacerbate endothelial damage.

Our study demonstrated a significant increase in ICAM-1 levels during ECMO, indicating sustained endothelial activation. This increase is consistent with previous findings that associate ICAM-1 with endothelial cell activation and inflammatory responses in critical illnesses [2]. Though not significant, weaning off ECMO seems to dampen this response again, as represented by a decrease in ICAM-1 after decannulation. Furthermore, the elevated ICAM-1 levels observed in non-survivors, especially at week 1, suggest a more pronounced inflammatory response in non-survivors compared to survivors. In contrast, the IL-6 levels decreased during ECMO and further declined after decannulation, indicating a potential resolution of systemic inflammation over time. These results are in line with a study in patients receiving VV- and VA-ECMO where non-survivors showed persistently high IL-6 levels compared to survivors [19]. The initial high levels of IL-6 in non-survivors compared to survivors highlight its role as a marker of disease severity and poor prognosis [20].

We show that VV-ECMO induces increases in endothelial activation and in vitro endothelial permeability, represented by changes in the endothelial angiopoietin/Tie2 system. Under physiological conditions, angiopoietin-1 activates the Tie2 receptor on endothelial cells, thereby stabilizing cell–cell junctions. When endothelial cells are activated, angiopoietin-2 is released from Weibel–Palade bodies. Angiopoietin-2, a context-dependent antagonist or agonist of Tie2, can then induce endothelial hyperpermeability [21,22]. In our cohort, the angiopoietin-1 levels significantly decreased after four days of ECMO and remained low. Interestingly, the angiopoietin-2 levels increased after ECMO initiation, with non-survivors showing particularly high levels after four days. The decrease in angiopoietin-1 and increase in angiopoietin-2 suggest a loss of vascular stabilization, which could contribute to increased permeability and tissue edema. The decrease in sTie2 during the first week of ECMO, followed by stabilization, may reflect an initial endothelial response to the ECMO-induced stress, with a subsequent adaptation over time. These results are in line with studies in patients on VA-ECMO who show a decrease in angiopoietin-1 and an increase in angiopoietin-2 during the first two days ECMO [23,24]. Furthermore, a previous study from our group has shown a similar increase in angiopoietin-2 after one week in patients with COVID-ARDS admitted to the ICU; however, concentrations were even higher in our patients on ECMO [18]. This might suggests a worsening effect of ECMO on endothelial barrier function, in addition to disease specific effects.

Interestingly, we observed that endothelial activation persisted during ECMO support, even though the inflammatory marker IL-6 showed a decline. However, other inflammatory markers such as TNF-α remained unchanged. Rather than a complete resolution of inflammation, our findings suggest a partial attenuation of the inflammatory response during ECMO support. Several mechanisms may explain the dissociation between systemic inflammatory markers and endothelial activation. Firstly, endothelial activation can be maintained by sustained exposure to non-inflammatory stressors, such as mechanical shear stress from the ECMO circuit [2]. Secondly, endothelial recovery may occur more slowly compared to the decline in inflammatory cytokines, particularly in the case of pre-existing endothelial injury due to the severe illness of these patients [25]. Lastly, persistent endothelial dysfunction and loss of endothelial barrier function have been described even after the resolution of systemic inflammation in patients with sepsis and viral pneumonia [26]. Taken together, these findings suggest that the endothelium may remain activated despite decreased systemic inflammation during ECMO support.

Syndecan-1 and thrombomodulin, markers of glycocalyx degradation, did not show significant changes during ECMO. In addition, levels prior to ECMO were not different from patients on MV. Nonetheless, both patient groups did show much higher concentrations of syndecan-1 and thrombomodulin compared to healthy volunteers, suggesting that glycocalyx degradation might have occurred in both groups earlier than the time points measured in our study. Our finding is consistent with previous studies reporting early glycocalyx degradation and elevated circulating levels of syndecan-1 in patients with severe COVID-19 [27,28,29]. This supports the hypothesis that extensive glycocalyx degradation likely occurred before ECMO was initiated, which may explain the absence of further increase during ECMO support in our study. It is possible that substantial glycocalyx damage already occurred due to the severity of the underlying viral disease, limiting the detection of additional ECMO-induced effects.

This exploratory study did not find changes over time regarding markers of hemolysis and hemostasis. As reported by the ELSO Guideline on Anticoagulation, hemolysis is an under-recognized complication of ECMO [10]. Surprisingly, we found a decrease in free hemoglobin levels after the initiation of ECMO, while levels remained stable during the following days on ECMO. The immediately decrease in CFHb might partially be explained by a great dilution factor of the blood due to the priming solution and resuscitation of the patient on ECMO. Thus, this might be a relative decrease rather than an absolute decrease in CFHb. During the ECMO run, concentrations of CFHb in our patient cohort are comparable to those found in previous studies [30,31]. Despite the stable levels of CFHb during the EMCO run, haptoglobin levels decreased and LDH levels increased over time but not significantly. The lack of significance might be due to the small sample size and exploratory character of this study. In addition, the significantly higher levels of haptoglobin in survivors after two days of ECMO suggest a beneficial role of haptoglobin in mitigating the effects of free hemoglobin.

In vitro endothelial barrier function was measured using ECIS, a technique used to quantify the real-time behavior of cells within a monolayer [17]. The resistance of the endothelial barrier was measured while stimulating the endothelial cells with patient plasma. In a previous study, our group demonstrated that the exposure of pulmonary endothelial cells to the plasma of patients with COVID-ARDS resulted in a decrease in in vitro endothelial barrier function [18]. The current study also shows the plasma from patients on VV-ECMO can induce in vitro pulmonary endothelial hyperpermeability, with more pronounced effects observed using the plasma obtained after four days and two weeks of ECMO. This may highlight the cumulative impact of the circulating markers associated with ECMO. One of the markers suggested to be involved in the disruption of the endothelial barrier function is angiopoietin-2, which also increased in our cohort [5,18]. However, future research should elucidate the role of different circulating markers in endothelial permeability during ECMO.

### Strengths and Limitations

This study has several limitations that should be acknowledged. Firstly, as an exploratory study with a small sample size, the statistical power for detecting subtle changes is limited. Furthermore, due to the small sample size, conclusions regarding subgroup analyses should be regarded as hypothesis-generating. Nonetheless, our findings provide a valuable initial overview of changes in circulating markers in a well-defined cohort of patients with COVID-ARDS receiving ECMO treatment. Secondly, our study population consisted exclusively of patients with COVID-19, which may limit the generalizability of our findings to patients on ECMO with other etiologies. Given the endothelial and inflammatory effects known to be associated with COVID-19, disease-specific pathophysiology may confound the observed results. Thirdly, an ideal control group was challenging to establish, although we mitigated this by using a within-subject control and by including a matched cohort of critically ill patients with COVID-ARDS for additional context. Fourthly, no data was available on the hematocrit of the patients, causing the potential under- or overestimation of certain markers. However, using samples from the Biobank, we were available to assess a broad panel of circulating markers across multiple time points. Lastly, we used relatively ‘healthy’ endothelial cells for our in vitro experiments, which may not have fully captured the complexities of endothelial dysfunction in patients on ECMO. Nonetheless, the representability of this in vitro model for assessing endothelial barrier function has been validated in previous studies [5,16,17,18].

## 5. Conclusions

This exploratory study showed that patients on VV-ECMO support due to severe COVID-ARDS have increased endothelial activation and damage over time but not inflammation. Interestingly, these findings were particularly present in non-survivors. Surprisingly, the markers of hemolysis were highest prior to the initiation of ECMO but remained stable thereafter. Furthermore, plasma from patients with COVID-19 on VV-ECMO support led to in vitro pulmonary endothelial hyperpermeability, with this effect becoming more pronounced using the plasma obtained at later time points during the ECMO run. Further research should explore the pathophysiological pathways leading to endothelial activation and damage in a larger cohort of patients on ECMO including different etiologies, thereby reducing organ failure in these critically ill patients.

## Figures and Tables

**Table 1:** Patient demographics.

	All Patients (n = 42)			VV-ECMO Patients (n = 14)			MV Patients (n = 28)		
			**n ^#^**			**n ^#^**			**n ^#^**
Male sex, n (%)	32	(76)	42	10	(71)	14	22	(79)	28
Age (years)	54	(46–62)	42	54	(45–61)	14	54	(50–62)	28
BMI (kg/m^2^)	27.8	(24.8–34.2)	42	27.3	(24.9–28.1)	14	31.6	(25.1–34.8)	28
BSA (m^2^)	2.00	(1.90–2.20)	42	1.95	(1.80–2.00)	14	2.05	(2.00–2.30)	28
Smoking, n (%)	1	(4)	26	0	(0)	9	1	(6)	17
Comorbidities, n (%)									
Diabetes mellitus II	35	(83)	42	12	(86)	14	23	(82)	28
Hypertension	32	(76)	42	12	(86)	14	20	(71)	28
SOFA score	6	(5-8)	42	5	(4-8)	14	8	(5-9)	28
ECMO duration (days)	-	-		15	(8–33)	14	-	-	0
ICU stay (days)	14	(8-30)	42	30	(10–52)	14	12	(8–18)	28
In-hospital mortality, n (%)	16	(38)	42	8	(57)	14	8	(29)	28

Data represent frequencies with percentage or median with interquartile range. # = deviating number of patients due to missing data. VV-ECMO = veno-venous extracorporeal membrane oxygenation, MV = mechanically ventilated, BMI = body mass index, BSA = body surface area, ICU = intensive care unit.

## Data Availability

The datasets used and/or analyzed during the current study are available from the corresponding author on reasonable request.

## Supplementary Materials

The following supporting information can be downloaded at: https://www.mdpi.com/article/10.3390/jcm14144866/s1, Supplementary File S1—Supplementary methods, Supplementary File S2—Propensity score matching, Supplementary File S3—ECMO vs. MV, Supplementary File S4—Survivors vs. non-survivors.

## Abbreviations

The following abbreviations are used in this manuscript:

ARDS	Acute respiratory distress syndrome
CFHb	Cell-free hemoglobin
COVID-19	Coronavirus disease 19
ECIS	Electric cell-substrate impedance sensing
ECMO	Extracorporeal membrane oxygenation
ELSO	Extracorporeal life support organization
HMVEC	Human microvascular endothelial cell
ICAM-1	Intracellular adhesion molecule 1
ICU	Intensive care unit
IL-6	Interleukin-6
LDH	Lactate dehydrogenase
LME	Linear mixed-effects model
MV	Mechanical ventilation
SMD	Standardized mean difference
sTie2	Soluble Tie2
TNFα	Tumor necrosis factor α
VV-ECMO	Veno-venous extracorporeal membrane oxygenation
vWF	von Willebrand factor
