# Supplementary material for: Endothelial Activation and Permeability in Patients on VV-ECMO Support: An Exploratory Study
Source: J Clin Med. 2025 Jul 9;14(14):4866. doi: 10.3390/jcm14144866 (PMC12295967; doi:10.3390/jcm14144866)
Supplement: Supplementary file 1 [file jcm-14-04866-s001.zip › Supplementary file 1 - Supplementary methods.pdf]

Supplementary file S1

Plasma analyses

- Luminex

The following markers were measured in all patient samples using a Luminex platform (Biotechne, Minneapolis, MN, USA) containing tumor necrosis factor α (TNF-α), intercellular adhesion molecule 1 (ICAM-1), E-selectin, P-selectin, angiopoietin-1, angiopoietin-2, soluble Tie2, syndecan-1, soluble thrombomodulin, von Willebrand Factor-A2 and D-dimer. Measurements were performed according to the manufacturer.

- Enzyme linked immunosorbent assay

Interleukin 6 (IL-6; R&D Systems, Minneapolis, MN, USA) and haptoglobin (AB108856, Abcam, Cambridge, UK) were measured using enzyme linked immunosorbent assay (ELISA) according to the manufacturer.

- Spectrophotometry

Activity of lactate dehydrogenase (LDH) was analyzed by spectrophotometry at 340 nm and 25 °C (18). Pyruvate was added to the sample as substrate after which the rate of NADH oxidation was measured.
CFHb in the plasma samples was determined using Kahn's algorithm (19). The absorbance of the samples was measured using the Spectramax M2e microplate reader (Molecular Devices, San Jose, CA, USA). Absorbances were measured at 562, 578 and 598 nm and the extinction values were used to calculate the concentration of CFHb.

Pulmonary endothelial cells

Human pulmonary microvascular endothelial cells (HMVEC-L, CC-2527, Lonza Group, Basel, Switzerland) were cultured on gelatin-coated flasks in complete medium at 37 °C in an atmosphere of 95% air and 5% CO_2. Complete medium (EGMTM-2 MV, Lonza Group, Basel, Switzerland) consisted of basal endothelial cell medium supplemented with fetal bovine serum, ascorbic acid, antibiotic and antimycotic supplement, hydrocortisone, fibroblast growth factor, epidermal growth factor, insulin-like growth factor, and vascular endothelial growth factor.

Electric-Cell Substrate Impedance Sensing

Resistance of endothelial cells was measured using electric cell-substrate impedance sensing (ECIS; Applied BioPhysics, Troy, NY, USA) as previously described (20-22). Passage six human pulmonary microvascular endothelial cells were transferred to gelatin-coated 96 wells ECIS culture plates pre-treated with 10mM L-cysteine (Merck, Darmstadt, Germany). After a confluent monolayer was formed, cells were exposed to 10% platelet-free plasma obtained from patients on VV-ECMO support. Each well contained plasma from one patient sample. Impedance of the endothelial monolayer was measured at 4000 Hz using ECIS software (210.0 PC; Applied Bio-Physics) until a steady-state was reached at 9 hours. All plasma samples were measured in duplo and normalized to baseline. Endothelial resistance was calculated using impedance measurements.

In vitro endothelial permeability

- Pulmonary endothelial cells

Human pulmonary microvascular endothelial cells (HMVEC-L, CC-2527, Lonza Group, Basel, Switzerland) were cultured on gelatin-coated flasks in complete medium at 37 °C in an atmosphere of 95% air and 5% CO_2. Complete medium (EGMTM-2 MV, Lonza Group, Basel, Switzerland) consisted of basal endothelial cell medium supplemented with fetal bovine serum, ascorbic acid, antibiotic and antimycotic supplement, hydrocortisone, fibroblast growth factor, epidermal growth factor, insulin-like growth factor, and vascular endothelial growth factor.

- Electric-Cell Substrate Impedance Sensing

Resistance of endothelial cells was measured using electric cell-substrate impedance sensing (ECIS; Applied BioPhysics, Troy, NY, USA) as previously described (18-20). Passage six human pulmonary microvascular endothelial cells were transferred to gelatin-coated 96 wells ECIS culture plates pre-treated with 10mM L-cysteine (Merck, Darmstadt, Germany). After a confluent monolayer was formed, cells were exposed to 10% platelet-free plasma obtained from patients on VV-ECMO support. Each well contained plasma from one patient sample. Impedance of the endothelial monolayer was measured at 4000 Hz using ECIS software (210.0 PC; Applied Bio-Physics) until a steady-state was reached at 9 hours. All plasma samples were measured in duplo and normalized to baseline. Endothelial resistance was calculated using impedance measurements.
