# Supplementary material for: Endothelial Activation and Permeability in Patients on VV-ECMO Support: An Exploratory Study
Source: J Clin Med. 2025 Jul 9;14(14):4866. doi: 10.3390/jcm14144866 (PMC12295967; doi:10.3390/jcm14144866)
Supplement: Supplementary file 1 [file jcm-14-04866-s001.zip › Supplementary file 2 - Propensity score matching.pdf]

Supplementary file S2

	1. No ECMO	2. ECMO	p
n	28	14	
gender = Male (%)	21 (75.0)	10 (71.4)	1.000
age (mean (SD))	53.80 (9.01)	53.50 (8.77)	0.919

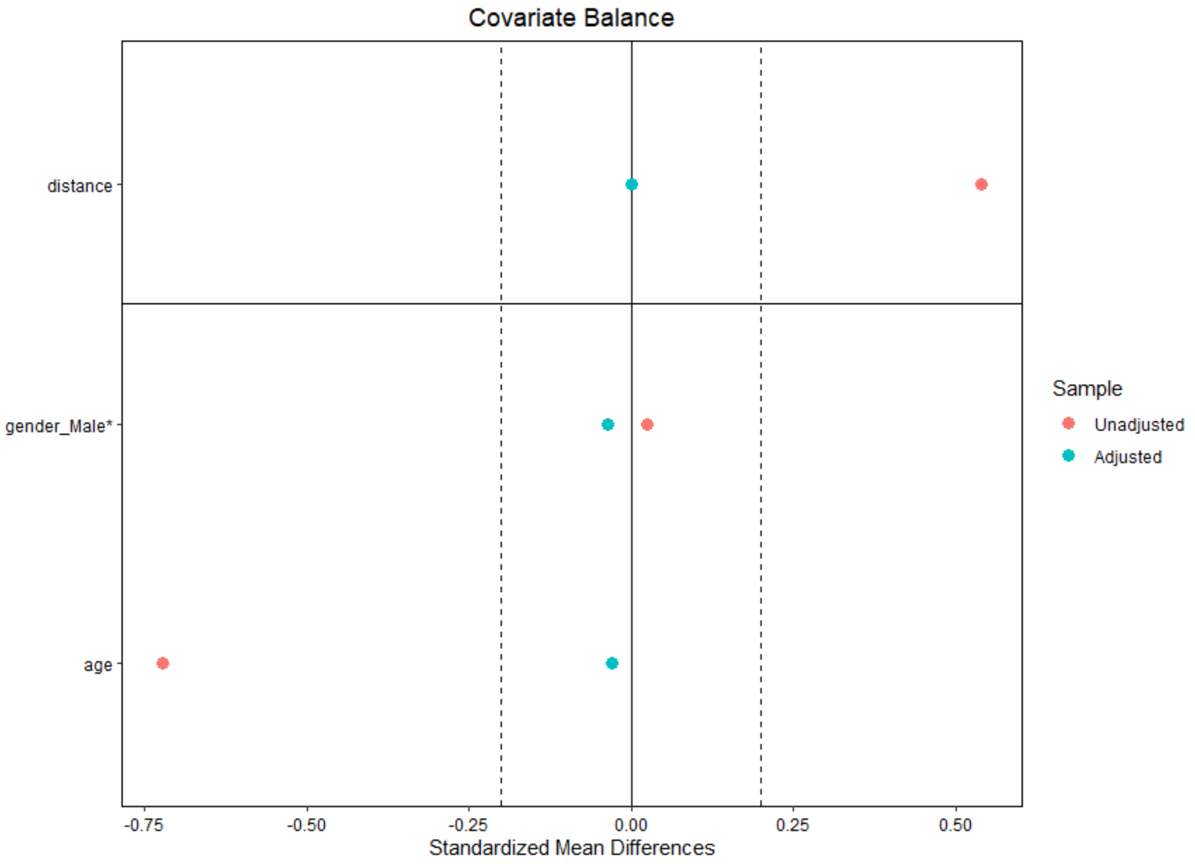

Supplementary figure S1 - Standard mean differences in the unadjusted and adjusted cohort after propensity score matching

Propensity score matching was considered successful and in balance since the SMDs were in between 0 and 0.2. Groups were defined as 'no ECMO' representing the mechanically ventilated patients and 'ECMO' representing t he VV-ECMO patients. ECMO = extracorporeal membrane oxygenation
