# Supplementary material for: Endothelial Activation and Permeability in Patients on VV-ECMO Support: An Exploratory Study
Source: J Clin Med. 2025 Jul 9;14(14):4866. doi: 10.3390/jcm14144866 (PMC12295967; doi:10.3390/jcm14144866)
Supplement: Supplementary file 1 [file jcm-14-04866-s001.zip › Supplementary file 3 - ECMO vs MV.pdf]

# Supplementary file S3

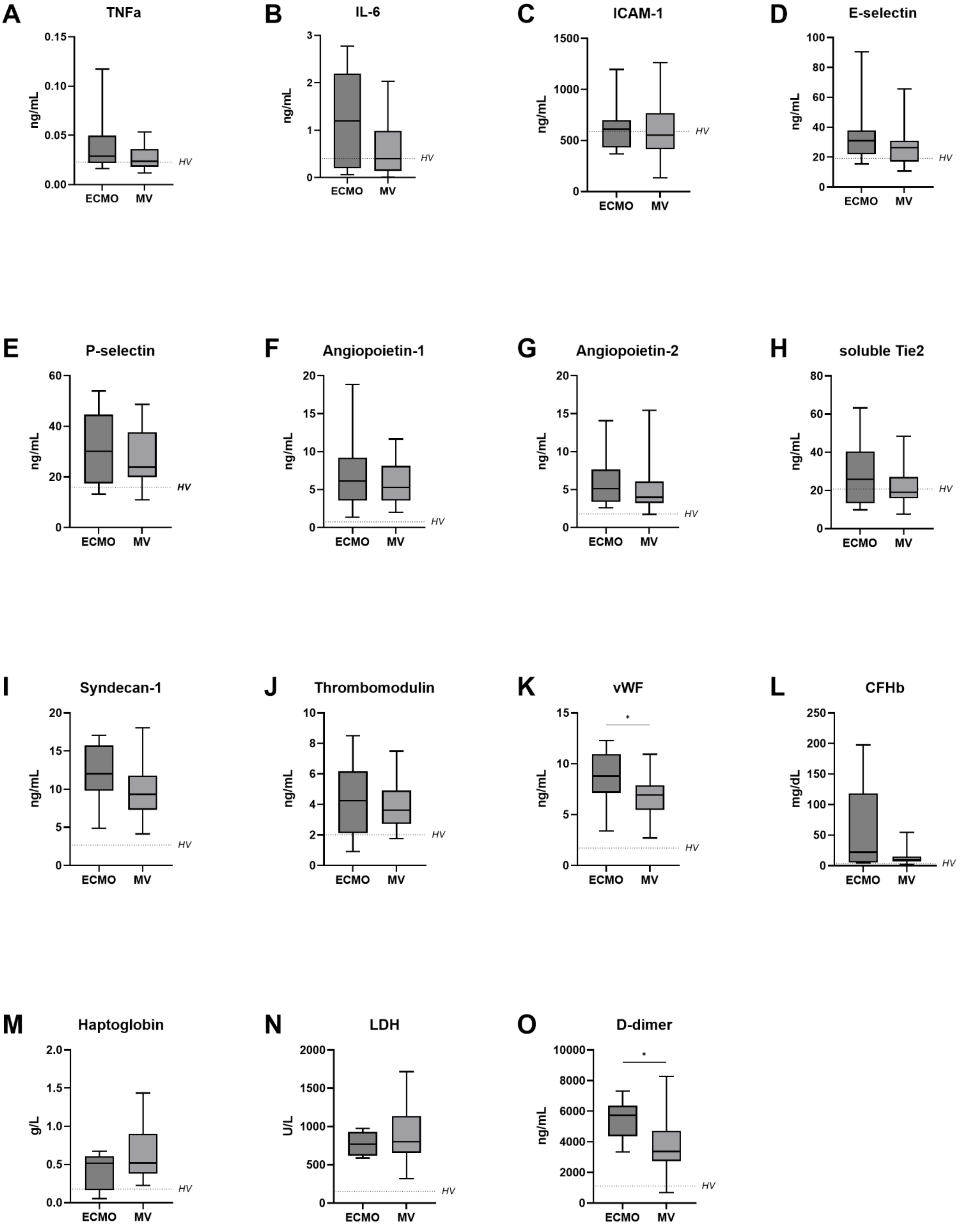

**Supplementary figure S2 – Circulating markers in patients prior to VV-ECMO and mechanically ventilated patients**

Circulating tumor necrosis factor α (TNFα; A), interleukin 6 (IL-6; B), intercellular adhesion molecule 1 (ICAM-1; C), E-selectin (D) and P-selectin (E), angiopoietin-1 (F), angiopoietin-2 (G), soluble Tie2 (H), syndecan-1 (I), thrombomodulin (J), von Willebrand Factor (vWF; K), cell-free hemoglobin (CFHb; L), haptoglobin (M), lactate dehydrogenase (LDH), and D-dimer (O) in plasma from VV-ECMO patients obtained before initiation of ECMO and mechanically ventilated patients at admission of the ICU. The dotted line represents mean values in healthy volunteers (HV). Data represent mean with standard deviation and were tested using mixed-effects models. * $p \leq 0.05$, ** $p \leq 0.01$, *** $p \leq 0.001$.
