# Supplementary material for: Endothelial Activation and Permeability in Patients on VV-ECMO Support: An Exploratory Study
Source: J Clin Med. 2025 Jul 9;14(14):4866. doi: 10.3390/jcm14144866 (PMC12295967; doi:10.3390/jcm14144866)
Supplement: Supplementary file 1 [file jcm-14-04866-s001.zip › Supplementary file 4 - Survivors vs non-survivors.pdf]

Supplementary file S4

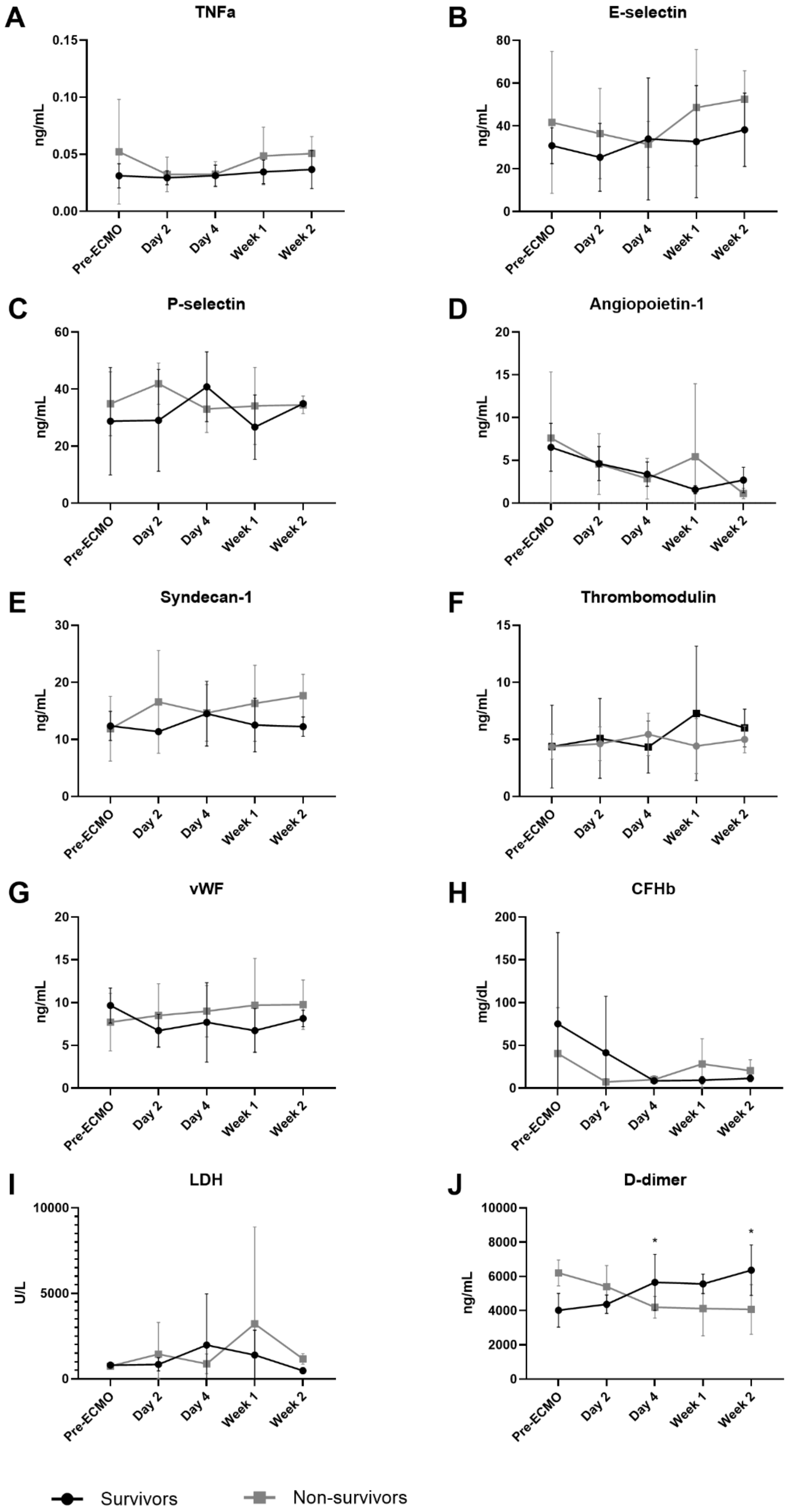

Supplementary figure S3 – Circulating markers in survivors and non-survivors of VV-ECMO

Circulating tumor necrosis factor α (TNF-α; A), E-selectin (B), P-selectin (C), angiopoietin-1 (D), syndecan-1 (E), thrombomodulin (F), von Willebrand Factor (vWF; G), cell-free hemoglobin (CFHb; H), lactate dehydrogenase (LDH; I), and D-dimer (J) in plasma from survivors and non-survivors of VV-ECMO obtained before initiation of ECMO (pre-ECMO), on day 2, day 4, week 1, and week 2 of ECMO support. Data represent mean with standard deviation and were tested using mixed-effects models. * $p \leq 0.05$, ** $p \leq 0.01$, *** $p \leq 0.001$.
